# Coracoclavicular Space Widening on Radiographs After Arthroscopic Stabilization With Suspensory Fixation Does Not Affect Athletic Performance

**DOI:** 10.1016/j.asmr.2021.01.005

**Published:** 2021-03-02

**Authors:** Amr Ibrahim, Saleh Gameel, Tarek Mohamed Ghandour, Begad M. Samy Abbas

**Affiliations:** a Orthopedic Department, Faculty of Medicine, Ain Shams University, Cairo, Egypt; b Physical Medicine, Faculty of Medicine, Ain Shams University, Cairo, Egypt

## Abstract

**Purpose:**

To evaluate the effect of the coracoclavicular distance widening in athletes after arthroscopic acromioclavicular (AC) stabilization using a suspensory button fixation device in terms of function, athletic performance, or isokinetic assessment.

**Methods:**

Sixty-eight athletes with a minimum 6-month follow-up after AC stabilization using suspensory button fixation were allocated in 2 groups, the widening group and non-widening group, according to the measured coracoclavicular distance after 6 months from the operation. The assessment was done every 6 months by Subjective Patient Outcome for Return to Sports (SPORTS) score, Athletic Shoulder Outcome Scoring System (ASOSS), Disabilities of the Arm, Shoulder, and Hand (DASH) score, Constant–Murley score (CMS), and the coracoclavicular distance. Isokinetic testing was performed at 24 months postoperatively to evaluate shoulder abduction and external rotation strength.

**Results:**

No statistically significant differences were found between the 2 groups in terms of the DASH, ASOSS, SPORTS, and the CMS, in addition to the isokinetic testing (*P* > .05). A statistically significant improvement in both groups over the follow-up stage was identified in the DASH, ASOSS, SPORTS, and the CMS (*P* < .05).

**Conclusions:**

Coracoclavicular distance widening following arthroscopic suspensory button fixation for AC joint dislocation did not affect function, athletic performance, or isokinetic evaluation in athletes.

**Level of Evidence:**

III; nonrandomized, comparative trial.

Acromioclavicular (AC) joint dislocations are a common injury that account for almost 50% of sports-related shoulder injuries.^1^, ^2^, ^3^ The Rockwood classification is the most commonly used classification system. Conservative management is widely used for type I and II dislocations, whereas surgical treatment the intervention of choice for type IV to VI dislocations.^1^^,^^4^, ^5^, ^6^

Many surgical options have been reported in the literature, all of which aim for full recovery with the return to the preinjury level of activity and sport participation.^6^, ^7^, ^8^ The suspensory button fixation was reported initially for the fixation of syndesmotic ankle injuries. The use of these devices in the treatment of AC dislocations has been described in open and arthroscopic techniques.^7^^,^^9^, ^10^, ^11^

The loss of AC joint reduction, as evident by coracoclavicular (CC) distance widening, is commonly reported after AC stabilization using suspensory button fixation devices. The effect of such findings on the athletic performance of patients is not yet fully understood.^7^^,^^9^^,^^11^, ^12^, ^13^, ^14^, ^15^ The purpose of this study is to evaluate the effect of CC distance widening in athletes after arthroscopic AC joint stabilization using a suspensory bottom fixation device in terms of function, athletic performance, and/or isokinetic assessment. We hypothesized that the CC distance rewidening after AC joint stabilization using a suspensory button fixation might affect the function and athletic performance of the shoulder joint.

## Methods

**Fig 1:**
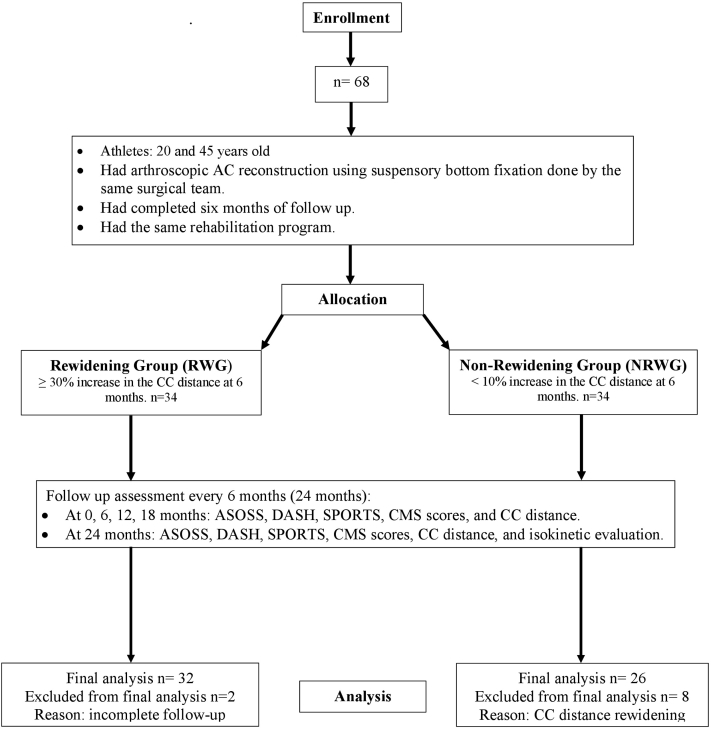
Study flowchart. (AC, acormioclavicular, ASOSS, Athletic Shoulder Outcome Scoring System; CC, Coracoclavicular; CMS, Constant–Murley Score; DASH, Disabilities of Arm, Shoulder and Hand; SPORTS, Subjective Patient Outcome for Return to Sports.)

The ethical committee approved this prospective study on March 2011. Between April 2011 and March 2016, we recruited 68 patients in this nonrandomized prospective cohort study (Fig 1), which was conducted at our institute. Eligible patients were atheletes aged between 20 and 45 years who had arthroscopic AC reconstruction using suspensory button fixation using the same suspensory button fixation device for acute AC dislocation Rockwood type III or V done within 1 month of the injury by our surgical team, using the same surgical technique described in our previous studies.^7^^,^^16^ All patients must have completed 6 months after the fixation and had the same rehabilitation program, which was formulated in 4 phases over 6 months, including (1) immobilization phase (weeks 0-6): during this phase, all patients were immobilized in a pouch arm sling; (2) intermediate phase (weeks 7-12): during this phase, active assisted and active range of motion were initiated along with stretching exercises; (3) strengthening phase (weeks 13-16); during this phase, strengthening exercises including isotonic strength activities were initiated; and (4) return to activity phase (months 4-6); during this phase, strength, balance and proprioception exercises, and sports-specific activities were started.

Subjects were included in the widening group (WG) if they had a 30% or more increase in the CC distance on the 6-month-follow up radiograph when compared with the immediate postoperative radiograph, which was collected retrospectively from the patients’ files. The non-widening group (NWG) included subjects with no or less than 10% increase in the CC distance at the same follow-up point. The CC distance was measured and recorded by 2 independent radiographers. To measure the CC distance, we used an anteroposterior view of the shoulder with AC projection (the beam was centered over the AC joint with 10° cephalad) that was done by the same technician. Seventeen subjects (10 in WG and 7 in NWG) were also participating in another study that was published in 2019.^7^^,^^9^, ^10^, ^11^

Subjects were excluded from the study if they were outside the age range, nonathletes, noncompliant to the rehabilitation program as seen retrospectively from their files, or if there was an increase in the CC distance of 10% or more but less than 30% of their immediate postoperative radiograph. Subjects also were excluded if they had any associated pathology of the shoulder, such as rotator cuff tear, shoulder instability, adhesive capsulitis, fracture or dislocation of the shoulder girdle, or previous shoulder surgery.

We considered patients as athletes if they had at least 2 training sessions each week. We classified the patients according to their level of sports participation in 3 grades: grade 1: professional athletes who consider sport as their primary source of income; grade 2: amateur athletes who had at least 3 training sessions per week; and grade 3: amateur athletes who had only 2 training sessions per week. Different types of sports were categorized into 4 subtypes, according to Allain et al.^17^ We included athletes from type III (swimming the crawl and tennis) and type IV (basketball, volleyball, handball, and karate).

The starting point of the study, at which the recruitment occurred, was 6 months from the operation. Participant were only recruited if they fulfilled the inclusion criteria. At recruitment, each subject was given a code; the code key was kept with the code keeper, who was nonmedical personnel. A baseline assessment was done for all the study subjects using (1) the Subjective Patient Outcome for Return to Sports (SPORTS) score^18^; (2) Athletic Shoulder Outcome Scoring System (ASOSS)^19^; (3) Disabilities of the Arm, Shoulder, and Hand (DASH) score^20^^,^^21^; (4) Constant–Murley score (CMS), which was done by a single experienced examiner in shoulder surgery; and (5) CC distance in the AC joint projection, which was measured and recorded by 2 independent radiographers to avoid intra- and interobserver bias. This baseline assessment was performed in all subjects at the time of recruitment, keeping in mind that subjects were recruited only if a 6-month duration had passed since the operation as a part of the inclusion criteria. All subjects in both groups were assessed every 6 months for 24 months using the aforementioned parameters.

### Isokinetic Evaluation

A single examiner (a physical medicine physician, M.D.) performed an isokinetic muscle strength evaluation for the shoulder external rotation and abduction in all patients at 24-month follow-up using the isokinetic Biodex System 3 (Biodex Medical Systems, Shirley, NY).

For assessment of external shoulder rotation, patients were positioned in a seated position with 45° of abduction in the scapular plane and 90° of elbow flexion. The rotational axis was aligned with the humeral shaft axis. Subjects were stabilized with straps, as described in the manufacturer’s manual.

For assessment of shoulder abduction, patients were positioned in a seated position with full elbow extension and full shoulder adduction as the starting position. The rotational axis was aligned with the axis of the AC joint. The nonoperated side was tested at the beginning to familiarize the patient with the procedure and was not included in the final analysis.

The test protocol consisted of concentric contractions at 3 angular speeds, 60° per second and 120° per second for 5 repetitions, and 180° per second for 10 repetitions. The test protocol was thoroughly explained to patients. A warm-up cycle preceded the initiation of the procedure, which consisted of a 5-minute upper body cycle of one maximal effort and three submaximal efforts.

Visual feedback was given, but not verbal encouragement. The normalized torque per body weight in Newton-meters per kilogram was measured. The results were collected, tabulated, and kept with the code keeper.

### Blinding

The clinical examiner, the 2 radiographers, and the physical medicine specialist were all blinded to the group allocation. The code keeper, who was nonmedical personnel, was responsible for keeping the study code key, the completed DASH, ASOSS, and SPORTS questionnaires, the CMS results, the radiographer’s reports. He was the only permitted researcher to approach for the patients by phone to schedule follow-up appointments and radiographic evaluations. The code keeper was not involved in the clinical assessment of patients.

### Confounders

As randomization was not possible in this study, we tried to control for possible confounders at the level of the study design by restriction and matching. Patients who were handled by different surgical teams or had different fixation methods, different follow-up duration, or were practicing extreme sports (martial arts, wrestling, or climbing, etc.) were not included in the study. We did not consider karate as an extreme sport, as we included 2 amateur karate players, one in each group. In addition, subjects in the NWG were matched to those in the WG in respect of age, sports type, and level of participation.

### Sample Size

We performed a 2-tailed a priori test evaluation for the needed sample size, to achieve power (1 – b) of 80% when alpha = 0.05, using the G∗Power software (version 3.1.9.6; Heinrich-Heine-Universität, Düsseldorf, Germany). The assessment was done for the CMS and the isokinetic evaluation of the shoulder abduction.

We assumed a mean CMS difference of 16 points between the 2 groups and a standard deviation of 20 points; 26 subjects had to be recruited in each group. For the isokinetic assessment of the shoulder abduction, we assumed a mean difference of 0.05 and a standard deviation of 0.06 Newton-meters per kilogram, 24 subjects had to be included in each group. Our target was to include 34 cases in each group in an attempt to compensate for lost study subjects during the follow-up.

### Statistics

We used the SPSS software (version 23.0; IBM, Armonk, NY) in the analysis of data. We calculated the range, mean, and standard deviation. The χ^2^ test was used to assess the statistically significant difference between the 2 groups in respect of the nominal variables. The intraclass correlation coefficient was calculated to measure the reliability of the readings of the 2 radiographers.

Age, sex, laterality, and type of sport and level of participation were identified as possible confounders. The multivariate analysis of covariance (MANCOVA) was conducted to test the effect of group allocation on the dependent variables while controlling for the age, sex, laterality, type of sport, and the level of participation. Before we conducted the MANCOVA test, data were tested for normality using the Kolmogorov–Smirnov test, homogeneity of variances and covariances using the Levene test, and homogeneity of regression slopes.

If the results of the MANCOVA test showed an insignificant effect of the suspected confounder, the independent sample *t* test was used to test numerical variables for statistically significant difference, and to calculate the mean difference and the 95% confidence interval of the mean difference.

When variables were measured for more than one occasion during the study, the 2-way repeated-measures analysis of variance (ANOVA) was used to detect statistically significant changes within the same group and to assess for any differences in the changes in relation to the time between the 2 groups.

Statistical significance was evaluated by the 95% confidence interval and the 2-tailed significance (*P* value). We rejected the null hypothesis if 95% confidence interval was narrow and did not include the zero value, in addition to a *P* value of < .05. A Bonferroni correction of *P* value was considered when multiple comparisons were used in the MANCOVA test; a statistically significant difference was considered if *P* value was < .03.

A minimal clinically important difference of 10.4 points for the CMS and 10.2 points for the DASH score was considered as cut points to consider a change in these scores as clinically significant.^22^^,^^23^

## Results

Sixty-eight subjects were recruited for this study. Thirty-four subjects were allocated in each group according to their CC distance. Only 58 patients were eligible for the final analysis (32 in the WG, and 26 in the NWG). Two cases from the WG were excluded from the final analysis for incomplete follow-up data due to noncompliance. In comparison, 8 cases from NWG were excluded from the final analysis, as CC distance widening occurred during the study course and after their recruitment.

The χ^2^ test revealed that there were no statistically significant differences between the 2 groups in terms of age, sex, laterality, sport type, or level of participation (Table 1). The intraclass correlation coefficient showed an acceptable reliability of the CC distance measurements between the 2 radiographers (Table 2).

**Table 1:** Study Demographics

	Widening Group (WG)	Non-widening Group (NWG)
Age, y	32.7 ± 8.48	35.6 ± 7.43
Sex		
Male	22	17
Female	10	9
Laterality		
Right	15	14
Left	17	12
Sport type		
Basketball	3	6
Volleyball	20	18
Handball	9	8
Swimming	10	9
Tennis	8	2
Karate	1	1
Participation Level		
Professional	3	3
Amateur: 3 sessions weekly	21	10
Amateur: 2 sessions weekly	8	13

**Table 2:** The ICC for the Reliability of the CC Distance Measurements

	ICC	Confidence Interval	
		Lower	Upper
CC pre	0.87	0.79	0.92
CC 0	0.89	0.82	0.94
CC 6	0.89	0.81	0.93
CC 12	0.93	0.88	0.95
CC 18	0.88	0.85	0.90
CC 24	0.86	0.84	0.88

NOTE. 0 indicates baseline assessment; 6, 6-month assessment; 12, 12-month assessment; 18, 18-month assessment; and 24, 24-month assessment.

CC, coracoclavicular; ICC, intraclass correlation coefficient.

The MANCOVA showed that there was no statistically significant effect of the age, sex, laterality, sports type, or level of participation on the results of the DASH, CMS, ASOSS, or SPORTS scores, *P* > .05. In addition, there was no statistically significant effect on the changes in the CC distance on the isokinetic muscle testing, *P* > .05 (Table 3).

**Table 3:** Effect of Confounders on the Statistical Difference Between the 2 Groups, Wilk`s Lambda test, MANCOVA

	F Value	*P*	Effect Size
Age	1.38	.222	.690
Sex	1.04	.473	.625
Laterality	.96	.551	.606
Sport type	1.07	.440	.633
Participation level	.57	.923	.478

MANCOVA, multivariate analysis of covariance.

Although the means of the DASH, CMS, and ASOSS scores, in addition to the isokinetic assessments, were greater in the NWG than in the WG, the independent sample *t* test showed that this difference was not statistically significant: in respect of the DASH score (at the recruitment; *P* = .568, at 6-month follow-up; *P* = .174, at 12-month follow-up; *P* = .073, at 18-month follow-up; *P* = .072, at 24-month follow-up; *P* = .089), in respect of the CMS score (at the recruitment; *P* = .066, at 6-month follow-up; *P*= .155, at 12-month follow-up; *P* = .090, at 18-month follow-up; *P* = .066, at 24-month follow-up; *P* = .097); in respect of the ASOSS score (at the recruitment; *P* = .107, at 6-month follow-up; *P* = .130, at 12-month follow-up; *P* = .064, at 18-month follow-up; *P* = .076, at 24-month follow-up; *P* = .080); in respect of the SPORTS score (at the recruitment; *P* = .215, at 6-month follow-up; *P* = .287, at 12-month follow-up; *P* = .392, at 18-month follow-up; *P* = .484, at 24-month follow-up; *P* = .785); in respect of the isokinetic assessments (external rotation at 60°/seconds of angular velocity; *P* = .067, external rotation at 120°/seconds of angular velocity; *P* = .072, external rotation at 180°/seconds of angular velocity; *P* = .083, abduction at 60°/seconds of angular velocity; *P* = .074, abduction at 120°/seconds of angular velocity; *P* = .071, abduction at 180°/seconds of angular velocity; *P* = .064). In addition, the 95% confidence intervals of the mean differences were wide and contained the zero value; therefore, we failed to reject the null hypothesis (Table 4).

**Table 4:** The Independent Sample *t* Test

	Widening Group (WG)			Non-widening Group (NWG)			Mean Difference	95% Confidence Interval of the Mean Difference		*P* Value
								Lower	Upper	
DASH 0	44.5	±	2.9	44.8	±	1.8	–3.8	–1.7	.95	.568
DASH 6	37.0	±	2.1	36.3	±	1.2	.64	–.29	1.5	.174
DASH 12	31.2	±	2.5	30.2	±	1.3	1.0	–.09	2.1	.073
DASH 18	24.1	±	2.5	23.0	±	1.5	1.0	–.09	2.1	.072
DASH 24	14.9	±	2.2	13.7	±	2.7	1.1	–.18	2.4	.089
CMS 0	64.2	±	4.1	67.1	±	6.5	–2.8	–5.7	–.03	.066
CMS 6	75.7	±	3.6	77.0	±	3.4	–1.3	–3.2	.5	.155
CMS 12	83.1	±	4.1	85.0	±	4.2	–1.9	–4.1	.3	.090
CMS 18	85.9	±	3.4	87.5	±	3.2	-1.6	–3.4	.1	.066
CMS 24	85.5	±	3.7	87.1	±	3.4	–1.5	–3.4	.3	.097
ASOSS 0	42.6	±	1.4	43.2	±	1.2	–.60	–1.3	.13	.107
ASOSS 6	49.8	±	2.9	50.8	±	1.9	–1.0	–2.3	.31	.130
ASOSS 12	57.0	±	2.8	58.2	±	1.5	–1.1	–2.4	.06	.064
ASOSS 18	66.7	±	4.7	68.5	±	1.9	–1.7	–3.7	.19	.076
ASOSS 24	78.7	±	4.7	81.1	±	5.8	–2.4	–5.2	.30	.080
SPORTS 0	3.0	±	.5	3.3	±	.97	–.25	–.65	.15	.215
SPORTS 6	3.2	±	.8	3.5	±	1.2	–.29	–.84	.25	.287
SPORTS 12	3.9	±	1.4	4.2	±	1.5	–.33	–1.1	.43	.392
SPORTS 18	5.8	±	2.2	6.3	±	2.7	–.46	–1.7	.85	.484
SPORTS 24	6.1	±	2.3	6.3	±	2.7	–.18	–1.5	1.1	.785
CC post	10.3	±	1.0	10.4	±	1.0	–.14	–.70	.40	.592
CC 0	14.9	±	.8	12.0	±	1.8	2.8	2.1	3.5	.000
CC 6	15.8	±	1.5	12.4	±	2.2	3.4	2.4	4.4	.000
CC 12	15.6	±	1.3	12.3	±	2.0	3.2	2.3	4.1	.000
CC 18	15.6	±	1.3	12.2	±	1.9	3.4	2.5	4.3	.000
CC 24	15.7	±	1.3	12.2	±	2.0	3.5	2.6	4.4	.000
External rotation 60°	.43	±	.02	.45	±	.03	–.01	–.03	.001	.067
External rotation 120°	.40	±	.02	.41	±	.04	–.01	–.03	.001	.072
External rotation 180°	.36	±	.02	.38	±	.03	–.01	–.03	.001	.083
Abduction 60°	.73	±	.01	.74	±	.01	.005	–.01	.001	.074
Abduction 120°	.71	±	.01	.72	±	.01	.006	–.01	.001	.071

NOTE. 0 indicates baseline assessment; 6, 6-month assessment; 12, 12-month assessment; 18, 18-month assessment; and 24, 24-month assessment.

ASOSS, Athletic Shoulder Outcome Scoring System; CC, Coracoclavicular; CMS, Constant–Murley Score; DASH, Disabilities of Arm, Shoulder and Hand; Post, immediately postoperative; SPORTS, Subjective Patient Outcome for Return to Sports.

**Fig 2:**
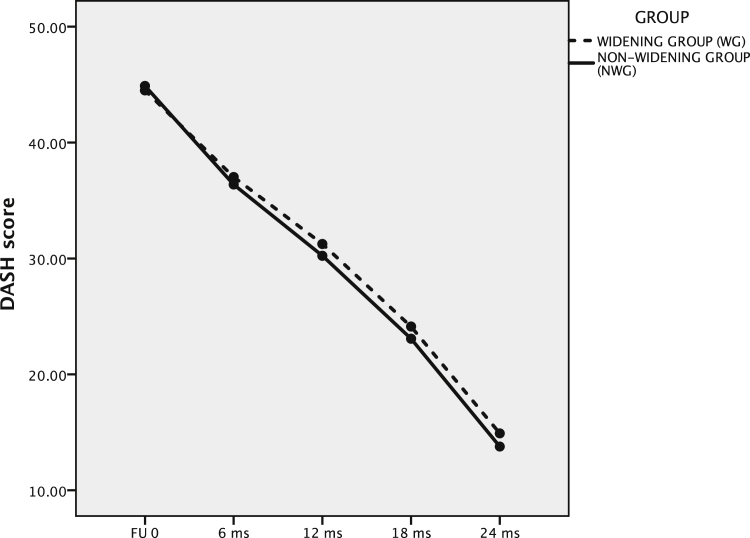
Changes in DASH score in relation to time in both groups. The graph shows improvement of the DASH score in both groups during the follow-up phase. (DASH, Disabilities of Arm, Shoulder and Hand.)

**Fig 3:**
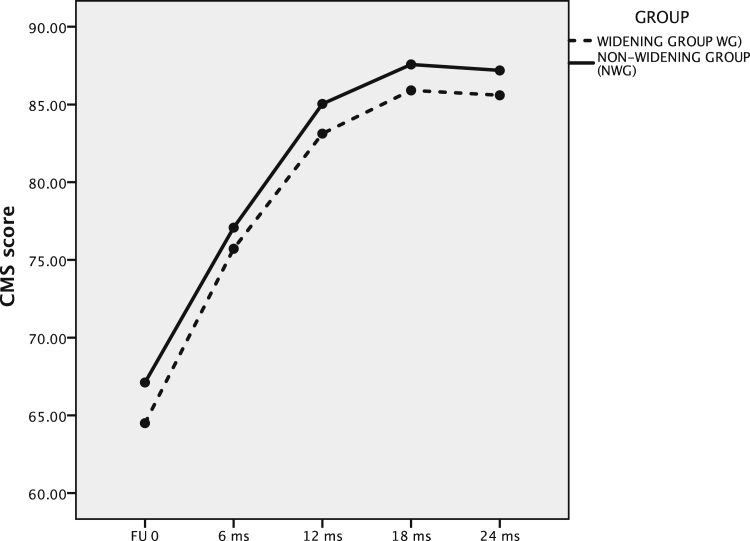
Changes in CMS in relation to time in both groups. The graph shows improvement in the CMS score in both groups during the follow-up phase. (CMS, Constant–Murley Score.)

**Fig 4:**
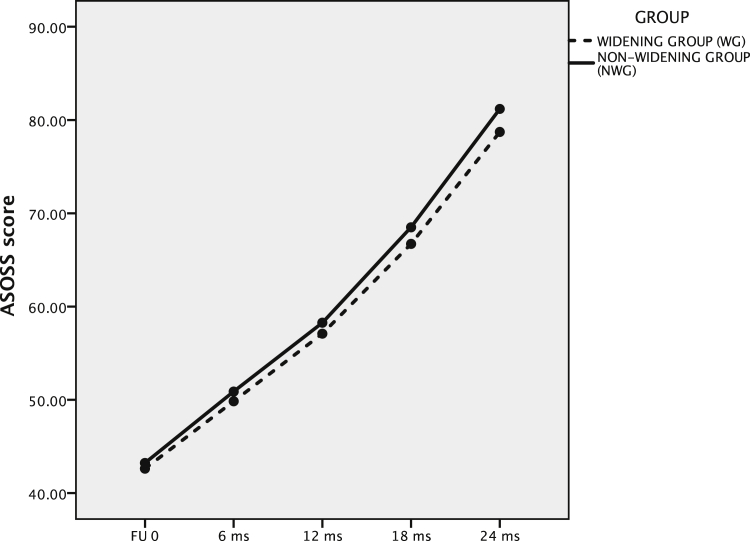
Changes in ASOSS in relation to time in both groups. The graph shows improvement in the ASOSS score in both groups during the follow up phase. (ASOSS, Athletic Shoulder Outcome Scoring System.)

**Fig 5:**
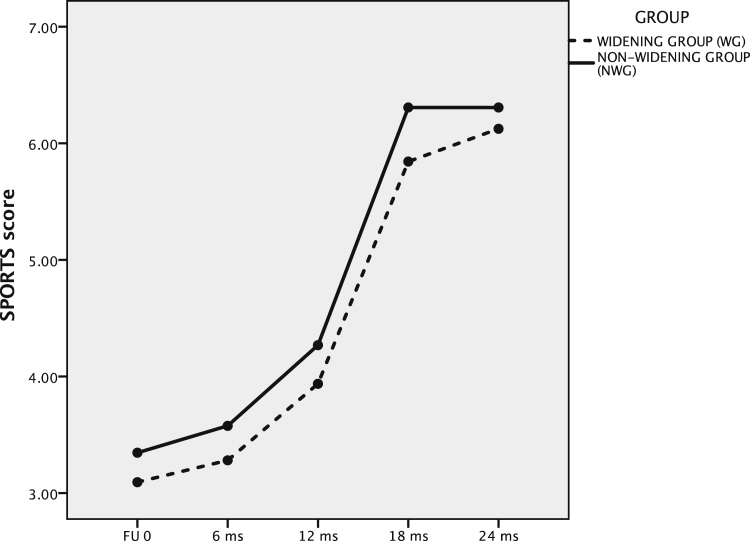
Changes in SPORTS in relation to time in both groups. The graph shows improvement in the SPORTS score in both groups during the follow-up phase. (SPORTS, Subjective Patient Outcome for Return to Sports.)

**Fig 6:**
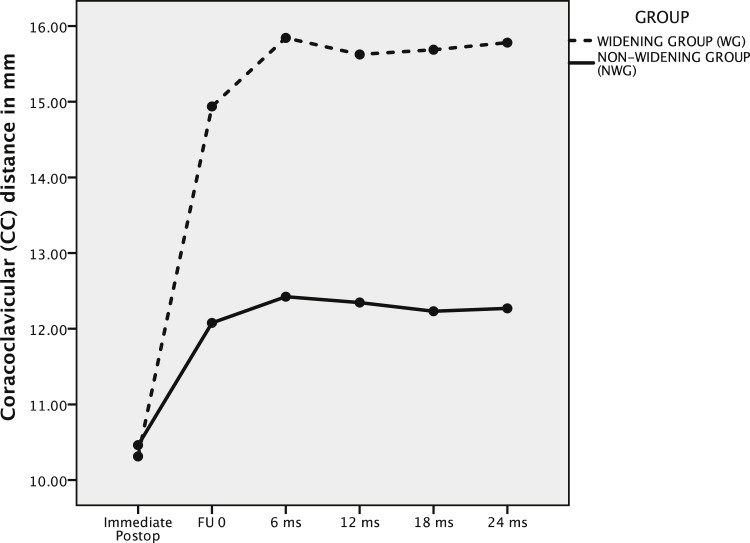
Changes in the CC distance in relation to time in both groups. The graph shows widening of the CC distance in the WG during the follow-up phase. (CC, coracoclavicular.)

The 2-way repeated-measures ANOVA yielded a significant improvement over the study course in the DASH, CMS, ASOSS, and SPORTS scores of each group (for DASH score; *P* = .515, for CMS score; *P* = .790, for ASOSS score; *P* = .663, for SPORTS score; *P* = .635). Therefore, the null hypothesis that there were no changes was rejected. The changes over time comparison did not show any significant differences between the 2 groups (*P* > .05). As expected, the 2-way repeated-measures ANOVA yielded a significant increase in the CC distance of the WG over the study course (*P* < .001), and nonsignificant changes in the NWG, (*P* > .05) (Fig 2, Fig 3, Fig 4, Fig 5, Fig 6).

In terms of clinical significance, all study subjects achieved the minimal clinically important difference for the CMS and the DASH scores from the initial stage to the final follow-up after 2 years.

## Discussion

We did not find statistically significant differences between the 2 groups in terms of function, athletic performance, or isokinetic assessment. We used the isokinetic testing as an objective tool to assess the shoulder function after suspensory button fixation of the AC joint reconstruction.

The loss of reduction after AC reconstruction using suspensory fixation devices has been reported frequently in the literature.^7^^,^^9^^,^^11^^,^^13^^,^^14^^,^^16^^,^^24^^,^^25^ While many researchers attempted to investigate the athletic performance following AC reconstruction, the effect of loss of reduction of the AC joint on the athletic performance of the patients was not reported in the literature before.^8^^,^^19^^,^^26^^,^^27^

In a systematic review done by Verstift et al.,^19^ they reviewed 12 articles that evaluated the return to the preinjury level of sport after a repair of Rockwood types III to VI dislocations, with a total number of patients of 498, of which 462 patients were engaged in sports activities. They concluded that the return to sport participation after this kind of surgery is high.

In another systematic review by Kay et al.,^27^ 315 patients, who had a surgical intervention for AC dislocation, were included in their reviewed articles. They concluded an excellent rate of sport participation after surgical reconstruction of the dislocated AC joint.

In addition, Saier et al.^15^ evaluated the return to activity level in 42 subjects after arthroscopically assisted reconstruction of acutely dislocated AC joint using suspensory button fixation. In their study, all included subjects were able to participate in sporting activities after the surgery. There was no observed difference in participation for those who practice contact or overhead sports.

In our study, all our 58 subjects returned to sport participation. Although we had greater results in the NWG in terms of function (CMS and DASH scores), athletic performance (ASOSS score), and the objective shoulder muscle strength (isokinetic testing), this difference was not statistically significant. This insignificant difference might have happened since we adjusted our sample size calculation to detect a power of 80%. A follow-up study with a greater power value, and eventually, a larger sample size might be needed to confirm any significant difference.

### Limitations

We had some limitations in our study; due to the nature of the study, we used a nonrandomized design, which might have increased the risk for confounders. We failed to reject the null hypothesis, even though we had greater results in the NWG. The definition of loss of reduction was based on a single point measurement at the time of recruitment. We defined widening as an increase of the CC distance of 30% or more, and non-widening as an increase of less than 10% at the time of recruitment. We excluded those with an increase of 10% to 30% to avoid this gray zone. During the follow-up phase, we had to exclude 8 subjects from the NWG due to a late-onset loss of reduction, which might have affected our final analysis.

## Conclusions

CC distance widening following arthroscopic suspensory button fixation for AC dislocation did not affect function, athletic performance, or isokinetic evaluation in athletes.

## Supplementary Data

ICMJE author disclosure forms
